# Neferine Ameliorates Sepsis-Induced Myocardial Dysfunction Through Anti-Apoptotic and Antioxidative Effects by Regulating the PI3K/AKT/mTOR Signaling Pathway

**DOI:** 10.3389/fphar.2021.706251

**Published:** 2021-07-22

**Authors:** Zhen Qi, Renrong Wang, Rongheng Liao, Song Xue, Yongyi Wang

**Affiliations:** ^1^ Department of Cardiovascular Surgery, Renji Hospital, School of Medicine, Shanghai Jiao Tong University, Shanghai, China; ^2^ Department of Cardiology, Wuxi No. 2 Hospital, Nanjing Medical University, Wuxi, China

**Keywords:** neferine, apoptosis, oxidative stress, mitochondria, lipopolysaccharide, cardiac dysfunction, sepsis

## Abstract

Septic cardiomyopathy is a common complication of severe sepsis, which is one of the leading causes of death in intensive care units. Therefore, finding an effective therapy target is urgent. Neferine is an alkaloid extracted from the green embryos of mature seeds of *Nelumbo nucifera* Gaertn., which has been reported to exhibit various biological activities and pharmacological properties. This study aims to explore the protective effects of neferine against lipopolysaccharide (LPS)-induced myocardial dysfunction and its mechanisms. The LPS-induced cardiac dysfunction mouse model was employed to investigate the protective effects of neferine. In this study, we demonstrated that neferine remarkably improved cardiac function and survival rate and ameliorated morphological damage to heart tissue in LPS-induced mice. Neferine also improved cell viability and mitochondrial function and reduced cell apoptosis and the production of reactive oxygen species in LPS-treated H9c2 cells. In addition, neferine significantly upregulated Bcl-2 expression and suppressed cleaved caspase 3 activity in LPS-induced mouse heart tissue and H9c2 cells. Furthermore, neferine also upregulated the phosphatidylinositol 3-kinase/protein kinase B/mechanistic target of rapamycin (PI3K/AKT/mTOR) signaling pathway *in vivo* and *in vitro*. Conversely, LY294002 (a PI3K inhibitor) reversed the protective effect of neferine in LPS-induced H9c2 cells. Our findings thus demonstrate that neferine ameliorates LPS-induced cardiac dysfunction by activating the PI3K/AKT/mTOR signaling pathway and presents a promising therapeutic agent for the treatment of LPS-induced cardiac dysfunction.

## Introduction

Sepsis is caused by the host’s inadequate immune response to infection, which can lead to life-threatening organ dysfunction (Singer et al., 2016). Septic cardiomyopathy is an acute myocardial injury induced by sepsis and characterized by impaired left ventricular systolic and diastolic functions (Beesley et al., 2018). The related mechanisms include inflammatory response, oxidative stress, calcium disorders, autophagy, apoptosis, and mitochondrial dysfunction (Hollenberg and Singer, 2021). Despite extensive advances in intensive care and support technology, septic cardiomyopathy is still the leading cause of death in non-coronary intensive care units (Merx and Weber, 2007; Levy et al., 2018; Wang et al., 2021). Although more aggressive approaches have been taken to prevent the progression of septic cardiomyopathy, these strategies are often disappointing, and the mechanisms underlying septic myocardiopathy remain unclear. Therefore, further elucidation of the mechanisms is crucial to finding an effective treatment for septic cardiomyopathy. Recent studies have demonstrated that herbal Chinese medicines are therapeutic against cardiovascular diseases. For example, Shikonin (extracted from Chinese herb radix arnebiae) ameliorated cardiac dysfunction and inhibited the activation of NLRP3 inflammasome in lipopolysaccharide (LPS)-induced cardiac dysfunction (Guo et al., 2020). Additionally, Songorinea (napelline-type C_20_-diterpene alkaloid in *Aconitum carmichaelii* Debx.) promoted cardiac mitochondrial biogenesis and inhibited oxygen free radicals in septic cardiomyopathy mice (Li et al., 2021).

Neferine is an alkaloid (molecular formula C38H44N2O6) extracted from the green embryos of mature seeds of *Nelumbo nucifera* Gaertn. It has been reported to exhibit various biological activities and pharmacological properties, including anti-inflammatory (Zhong et al., 2020), anti-arrhythmic (Qian, 2002), anti-platelet aggregation (Yang et al., 2019), antioxidant (Wu et al., 2019), anti-hypertensive (Wicha et al., 2020), and anti-atherosclerotic effects (Zhou et al., 2013), and confer a protective effect against hypoxia-induced oxidative stress by reactive oxygen species (ROS) scavenging and prevention of NF-kB nuclear translocation (Baskaran et al., 2016). In addition, neferine plays an important role in doxorubicin-treated H9c2 cardiomyoblasts, as shown by increased H9c2 cell viability, inhibited mitochondrial superoxide generation, and reduced inflammatory response (Priya et al., 2017; Bharathi Priya et al., 2018). However, no studies have reported the effects and molecular mechanisms of neferine against LPS-induced cardiac dysfunction, to the best of our knowledge. The phosphatidylinositol 3-kinase (PI3K)/protein kinase B (AKT) pathway can be activated by external stimuli to phosphorylate the downstream signaling molecule AKT, then phosphorylate the downstream signaling molecule mechanistic target of rapamycin (mTOR), thereby regulating a wide variety of biological responses, including inflammation, cellular proliferation, autophagy, and apoptosis, all of which may be involved in cardiac disease (Wang et al., 2019). Therefore, elucidating the signaling pathways activated by neferine could identify a potential therapeutic target for septic cardiomyopathy.

The aim of this study was to examine whether neferine had a cardioprotective effect against septic cardiomyopathy and to explore the related molecular mechanisms. Our results indicate that neferine treatment significantly reduced cardiomyocytes apoptosis, improved cardiac dysfunction, and ameliorated ROS production. These beneficial effects are most likely mediated by upregulating the PI3K/AKT/mTOR signaling pathway. Collectively, these findings indicate the efficacy of neferine treatment for septic cardiomyopathy.

## Materials and Methods

### Reagents

**FIGURE 1:**
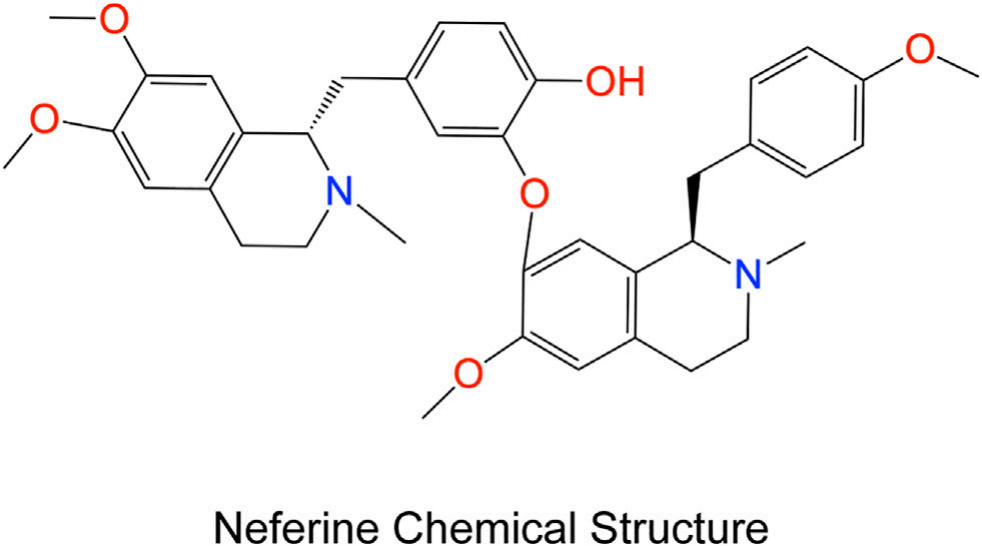
The chemical structure of neferine.

Neferine (purity ≥99%) and LY294002 (purity ≥99%) were purchased from MedChemExpress (Brea, CA, United States ). The structure of neferine is shown in Figure 1. LPS (L2880) was bought from Sigma (St. Louis, MO, United States).

### Animals

All animal-related experimental procedures were approved by the Animal Care and Use Committee of Renji Hospital, School of Medicine, Shanghai Jiao Tong University. Male C57BL/6J mice aged 6–8 weeks (20–24 g) were purchased from the Institute of Laboratory Animal Science, Chinese Academy of Medical Sciences (Shanghai, China). The mice were housed in constant temperature (24°C ± 2°C) and humidity (50–60%) under a 12-h light/dark cycle with free access to standard food and water. Mice were randomly divided into four groups as follows: control (Ctrl group), neferine (Nef group), septic myocardial injury (LPS group), and neferine combined with LPS (LPS + Nef group).To establish the septic myocardial injury model, mice were intraperitoneally injected with 10 mg/kg LPS (Liu et al., 2021). In the Ctrl group, the same amount of normal saline was injected intraperitoneally. The LPS + Nef group were injected intraperitoneally with 20 mg/kg neferine 2 h before LPS injection, and the Nef group were injected with 20 mg/kg neferine as described previously (Liu et al., 2018). After 12 h, the mice were sacrificed. Serum and heart tissue were collected and stored at −80°C for subsequent experiments. To measure mortality rate, mice were intraperitoneally injected with 15 mg/kg LPS. Neferine (20 mg/kg) was intraperitoneally administered 2 h before LPS challenge and then administered for three consecutive days. The mortality was recorded for 72 h.

### Echocardiography

After 12 h of LPS injection, echocardiography was performed using a high-resolution imaging system for small animals (Vevo 3,100 Imaging System, Visual Sonics, Japan). Heart rate (HR), fractional shortening (FS), ejection fraction (EF), left ventricular internal systolic dimension (LVIDs), left ventricular internal diastolic dimension (LVIDd), left ventricular posterior wall systolic thickness (LVPWs), and left ventricular posterior wall diastolic thickness (LVPWd) were analyzed using Vevo3100 software (Visual Sonics).

### Cell Culture and Modeling

H9c2 cells were procured from Stem Cell Bank, Chinese Academy of Sciences and maintained in Dulbecco’s modified Eagle medium supplemented with 10% fetal bovine serum and antibiotics (100 U/mL penicillin, 100 μg/ml streptomycin) at 37°C with 5% CO_2_ and 95% O_2_. Cells were grown on 6-well culture dishes until 70–80% confluence. To examine the protective effects of neferine on apoptotic damage and oxidative stress, H9c2 cells were initially preincubated with neferine (2 μM) for 24 h. After refreshing the medium, the cells were treated with LPS (10 μg/ml) for 12 h to create the LPS-induced cardiac injury cell model. The cells were divided into six groups: Ctrl group, Nef group, LPS group, LPS + Nef group, LY294002 group, and LPS + Nef + LY294002 group. Cells were collected for further analysis.

### Cell Counting Kit-8 Assay (CCK 8)

Cell viability was evaluated using the Cell Counting Kit-8 (CCK 8) Assay Kit (Vazyme, China). 5 × 10^3^ H9c2 cells/well were cultured in 96-well plates and incubated overnight. After the cells achieved 70% confluence, they were incubated with different LPS or neferine concentrations (5, 10, 20, and 50 μg/ml) for 24 h. Subsequently, 10 μl CCK-8 solution was added to each well. After incubation at 37°C for 2 h, the absorbance was measured at 450 nm.

### Measurement of the Mitochondrial Membrane Potential (ΔΨm)

The mitochondrial membrane potential (MMP) of H9c2 cells was evaluated by staining with JC-1, an MMP-specific fluorescent dye. The JC-1 Mitochondria Membrane Potential Assay Kit was obtained from Beyotime Biotechnology (Shanghai, China). When the mitochondrial membrane potential is high, JC-1 aggregates in the matrix of the mitochondria to form a polymer (J-aggregates), which produces red fluorescence. When the mitochondrial membrane potential is low, JC-1 cannot aggregate in the matrix of mitochondria and produces green fluorescence as a monomer. This provides a convenient way to detect the change of mitochondrial membrane potential through the change of fluorescence color. All experimental procedures were performed according to the manufacturer’s instructions. Briefly, H9c2 cells were washed twice with phosphate buffer saline (PBS), and then incubated with JC-1 for 30 min at 37°C, covered to prevent exposure to light. After washing twice with JC-1 washing buffer, images were captured by fluorescence microscopy. Red fluorescence means the normal mitochondrial potential, whereas green fluorescence indicates damaged mitochondrial potential. The red-to-green ratio of fluorescence intensity was used to evaluate the mitochondrial membrane potential. The relative fluorescence intensity was measured using Image J software (NIH, MD, United States).

### Intracellular ROS Determination

The intracellular ROS levels of H9c2 cells were determined using the ROS Assay Kit (Beyotime Biotechnology). Briefly, H9c2 cells were plated in 6-well plates and pretreated with neferine at 2 μM for 24 h before incubating with LPS for 12 h. Then, 2–7′ dichlorodihydrofluorescein diacetate (DCFH-DA; 10 μM) was added to the wells for 20 min at 37°C in the dark. The cells were observed by fluorescence microscopy. The relative intensity of DCFH-DA fluorescence was measured using Image J software.

### Terminal Deoxynucleotidyl Rransferase dUTP Nick End Labeling Fluorescent Staining

Terminal deoxynucleotidyl transferase (TdT) dUTP nick end labeling (TUNEL) was determined using the TUNEL Apoptosis Assay Kit (Beyotime Biotechnology) *in vivo* and *in vitro*, according to the manufacturer’s instructions. The H9c2 cells were cultured in 12-well plates, washed twice with PBS, and fixed in 4% paraformaldehyde for 30 min. After immersing in 0.3% Triton X-100 for 5 min, the cells were stained with 50 μl TdT solution and 450 μl fluorescein-labeled dUTP solution for 60 min at 37°C in the dark. Cells were washed and incubated with 4′,6-diamidino-2-phenylindole (DAPI) for 15 min.Cells were then analyzed for positivity by fluorescence microscopy at 450–550 nm. Blue fluorescence (DAPI) labeled the cell nuclei, whereas green fluorescence (TUNEL) labeled the apoptotic cells. The TUNEL-positive cell count is the ratio of the number of cells labeled by TUNEL to the number of cells labeled by DAPI.

### Hematoxylin and Eosin (H and E) Staining and Heart Injury Score

The heart tissues were fixed in 4% paraformaldehyde, embedded in paraffin, and sectioned into 5-μm slices. For morphological analysis, the paraffin sections were stained with hematoxylin and eosin (H and E) solution (Beyotime Biotechnology) and analyzed under an optical microscope. Myocardial edema and the numbers of inflammatory cell infiltration, myocardial fiber rupture, and necrotic cells were determined. Heart tissue sections were evaluated as previously described (Braun et al., 2017) using the scoring system explained subsequently to grade the degree of heart injury. The severity of myocardial tissue damage was assessed using pathological score = edema score + necrosis score + neutrophilic infiltration score + myocardial fiber rupture score. Five slices were evaluated in each group.

### Western Blot Detection

H9c2 cells and heart tissue lysates were harvested by radioimmunoprecipitation assay (RIPA) lysis (Beyotime Biotechnology) supplemented with phenylmethylsulfonyl fluoride and a phosphatase inhibitor, and the concentration was measured using the BCA Protein Assay Kit (Thermo Fisher Scientific, United States). An equivalent amount of protein was separated by sodium dodecyl sulfate–polyacrylamide gel electrophoresis and then transferred to polyvinylidene fluoride membranes (Millipore, United States). After blocking with 5% non-fat milk in Tris-buffered saline with Tween 20 at room temperature for 1 h, the membranes were incubated with primary antibodies at 4°C overnight. After that, they were probed with the corresponding horse-radish peroxidase-conjugated secondary antibodies (Beyotime Biotechnology) for 1 h at room temperature. For immunoreactive detection, enhanced chemiluminescence (ECL, Vazyme, China) was performed, and images were analyzed using Image J software. The following primary antibodies were utilized at 1:1,000 dilution: cleaved caspase 3 (Cell Signaling Technology, United States), Bcl-2 (Abcam, United Kingdom), Tubulin (Cell Signaling Technology), GAPDH (Cell Signaling Technology), P-PI3K (Cell Signaling Technology), PI3K (Cell Signaling Technology), P-AKT (Cell Signaling Technology), AKT (Cell Signaling Technology), P-mTOR (Cell Signaling Technology), mTOR (Cell Signaling Technology), SOD2 (Abcam, United Kingdom), SOD1 (Santa Cruz, United States), and iNOS (Abcam, United Kingdom). The detailed antibodies information in our research were given in Table 1.

**TABLE 1:** Antibodies used for protocols. Cat.-No: catalogue number of manufacturer; pc: polyclonal; mc: monoclonal; WB: western blotting.

Target	Product/Cat.-No	Origin	Clonality	Protocol/Dilution
Cleaved-caspase 3	Cell signaling technologies; # 9664S	Rabbit	Mc	WB (1:1,000)
Bcl-2	Abcam; ab182858	Rabbit	Mc	WB (1:1,000)
p-PI3K	Cell signaling technologies; # 17366S	Rabbit	Mc	WB (1:1,000)
p-AKT	Cell signaling technologies; # 4060S	Rabbit	Mc	WB (1:1,000)
P-mTOR	Cell signaling technologies; # 5536S	Rabbit	Mc	WB (1:1,000)
PI3K	Cell signaling technologies; # 4255S	Rabbit	Mc	WB (1:1,000)
AKT	Cell signaling technologies; # 4691S	Rabbit	Mc	WB (1:1,000)
mTOR	Cell signaling technologies; # 2983S	Rabbit	Mc	WB (1:1,000)
SOD1	Santa cruz; sc-101523	Mouse	Mc	WB (1:1,000)
SOD2	Abcam; ab68155	Rabbit	Mc	WB (1:1,000)
iNOS	Abcam; ab178945	Rabbit	Mc	WB (1:1,000)
Tubulin	Cell signaling technologies; # 2148S	Rabbit	Mc	WB (1:2,000)
GAPDH	Cell signaling technologies; # 5174S	Rabbit	Mc	WB (1:5,000)

### Intracellular Adenosine Triphosphate Assay

Intracellular adenosine triphosphate (ATP) levels were determined using the Enhanced ATP Assay Kit (Beyotime Biotechnology). Briefly, the cells were lysed, then centrifuged at 4°C and 12,000 *g* for 5 min. After centrifugation, the supernatant was collected. Detecting solution was added to a 96-well plate incubated at room temperature in the dark for 5 min. After incubation, the supernatant was added to the plate. The total ATP levels were analyzed by luminescence and normalized by the protein concentrations.

### Cardiac Troponin Assay

Blood was collected and centrifuged at 3,000 rpm for 15 min. The supernatant was collected. The cardiac troponin (cTnI) levels in serum were determined using the cTnI ELISA Kit (Lengton, China) according to the manufacturer’s instructions.

### Statistical Analysis

All data were presented as mean ± standard deviation (SD). Comparisons between the two groups were performed using Student’s t-test. Comparisons between multiple groups were performed using one-way analysis of variance. For survival analysis, the Kaplan–Meier survival curve was used with a log-rank test. *p* values <0.05 were considered statistically significant, and statistical analyses were performed with GraphPad Prism 8.0.

## Results

### Neferine Improved Survival Rate and Cardiac Function in Septic Mice

**FIGURE 2:**
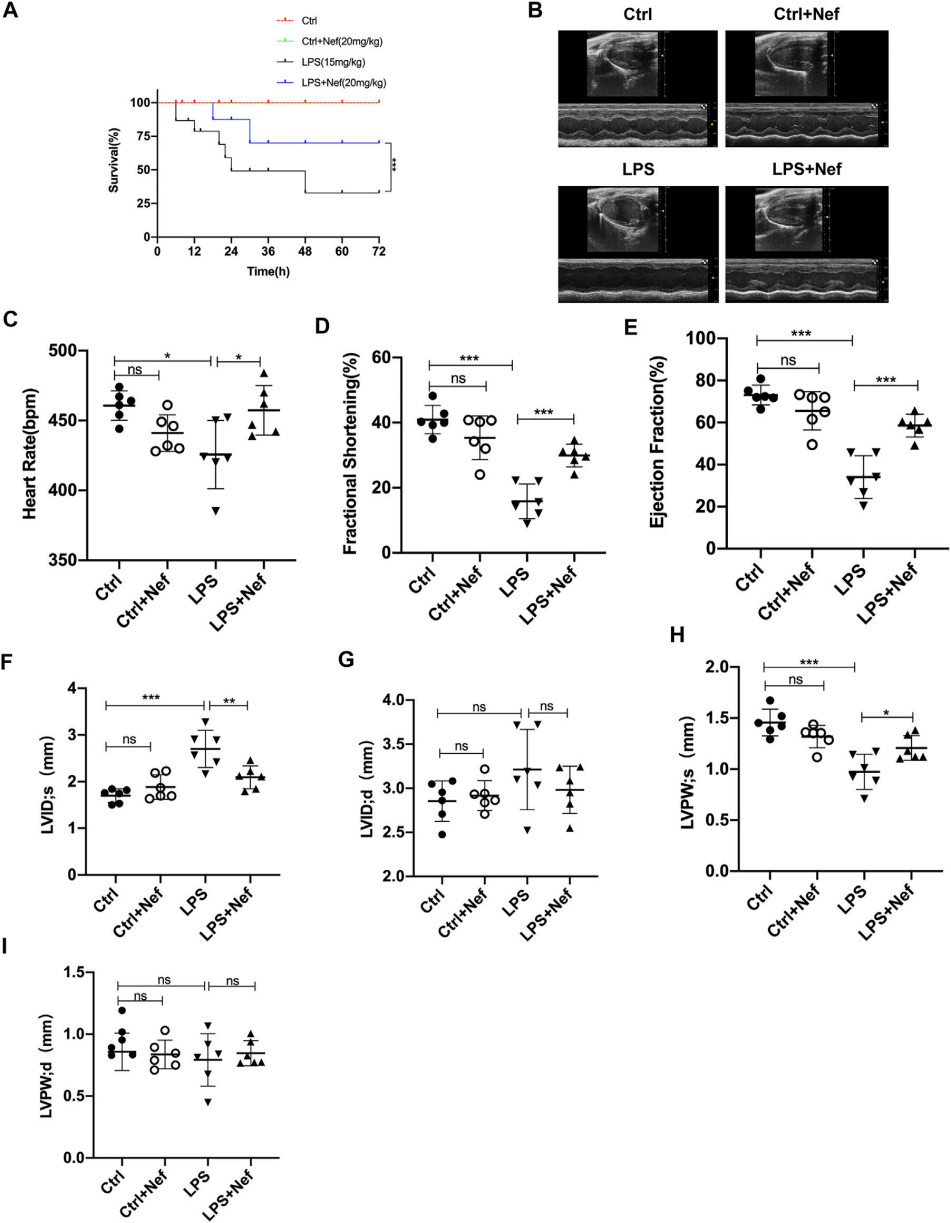
Neferine preserved cardiac function and improved the survival rate in LPS-treated mice **(A)** Neferine (20 mg/kg) was intraperitoneally administered 2 h before LPS injection (15 mg/kg) and then administered for three consecutive days. The mortality of mice within 72 h was recorded (n = 15 mice) **(B–I)** the mice were treated with neferine (20 mg/kg, intraperitoneally (i.p.) 2 h before LPS challenge (10 mg/kg, i.p.), and cardiac function was examined (n = 6) **(B)** Representative echocardiographic images **(C)** Heart rate (HR) **(D)** Fractional shortening (FS) **(E)** Ejection fraction (EF) **(F)** Left ventricular internal systolic dimension (LVIDs) **(G)** Left ventricular internal diastolic dimension (LVIDd) **(H)** Left ventricular posterior wall systolic thickness (LVPWs) **(I)** Left ventricular posterior wall diastolic thickness (LVPWd). Data are expressed as mean ± standard deviation. *p < 0.05, **p < 0.01, ***p < 0.001; ns: no significant difference.

The 72-h mortality of the LPS group was 67%, whereas the LPS + Nef group was 30%, which showed prolonged survival (Figure 2A). To investigate the effects of neferine on cardiac function in LPS-induced sepsis mice model, cardiac function parameters were evaluated by echocardiography (Figure 2B). The echocardiography results revealed that LPS-treated mice had significantly impaired cardiac function, as evidenced by the markedly reduced HR (Figure 2C), FS% (Figure 2D), EF% (Figure 2E), and LVPWs (Figure 2H) and markedly increased LVIDs (Figure 2F). Neferine treatment markedly reversed these adverse effects. No significant differences were found in LVIDd (Figure 2G) and LVPWd (Figure 2I) among the groups. These results demonstrate that neferine treatment protected cardiac function and improved the survival rate in LPS-induced cardiomyopathy mice.

### Neferine Prevented Myocardial Injury and Apoptosis in Septic Mice

**FIGURE 3:**
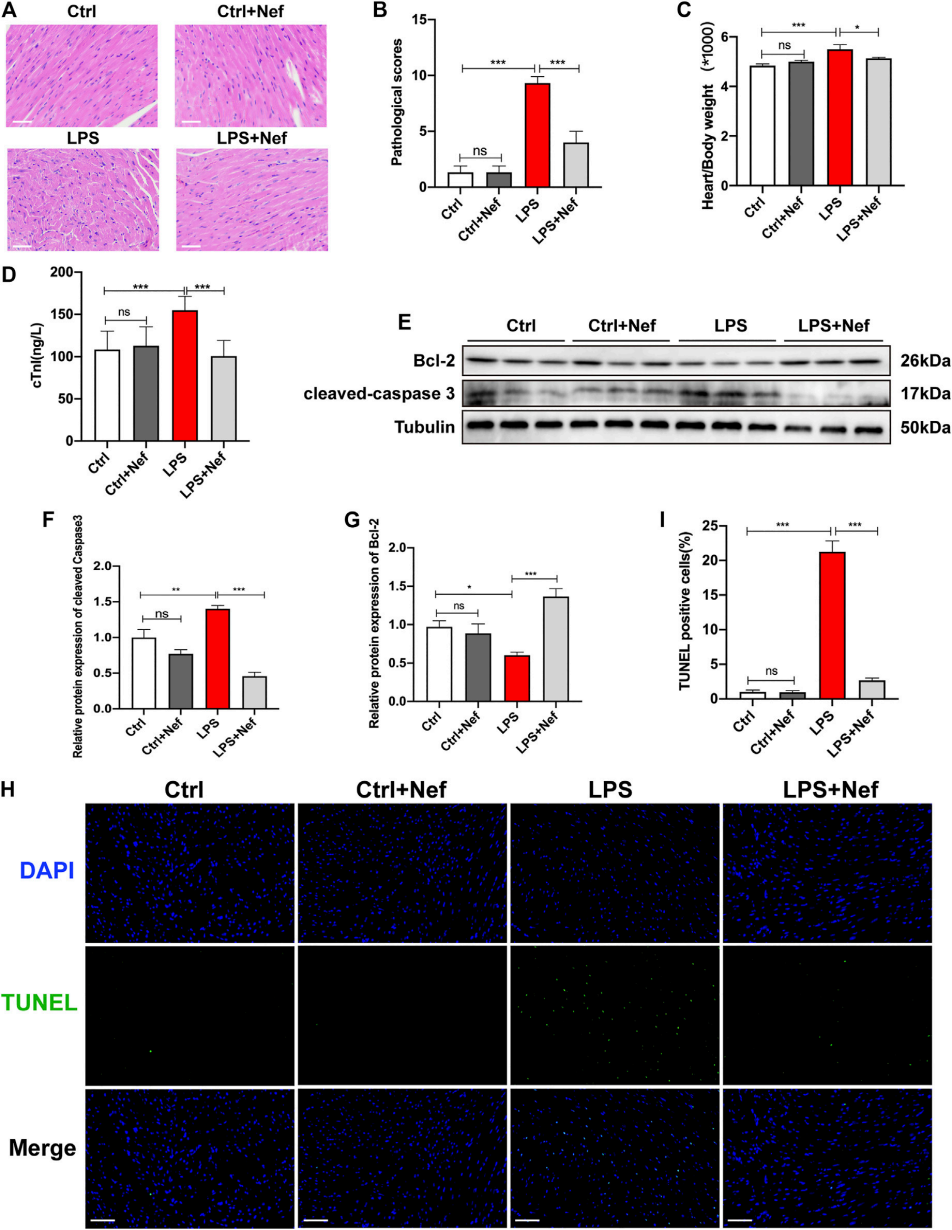
Neferine prevented myocardial injury and apoptosis in septic mice **(A)** Hematoxylin and eosin staining (n = 6 mice; Scale bar, 50 μm) **(B)** Pathological score of heart injury **(C)** Heart weight/body weight ratio (HW/BW; n = 10) **(D)** Serum cardiac troponin (cTnI) levels detected by ELISA (n = 6) **(E)** Protein expression levels of cleaved caspase 3 and Bcl-2 detected by Western blot (n = 6) **(F–G)** Densitometric quantification of the protein expression levels of cleaved caspase 3 and Bcl2 **(H)** Representative images of TUNEL staining of mice heart tissue. Scale bar, 50 μm **(I)** Measurement of TUNEL-positive cell ratio. Data are expressed as mean ± standard deviation. *p < 0.05, **p < 0.01, ***p < 0.001; ns: no significant difference; ELISA, enzyme-linked immunosorbent assay; TUNEL, terminal deoxynucleotidyl transferase dUTP nick end labeling.

Extensive inflammatory damage of cardiomyocytes is the basic pathologic feature of sepsis myocardiopathy. We investigated the effects of neferine on cardiomyocytes in LPS-induced myocardial dysfunction. H and E staining showed that neferine pretreatment significantly inhibited histological alterations including interstitial edema, myocardial fiber rupture, and inflammatory cell infiltration in heart tissue sections of LPS-treated mice (Figure 3A,B). In addition, neferine also reduced the heart weight/body weight ratio **(** Figure 3C) and reversed the LPS-induced elevation of serum cardiac troponin (cTnI) levels (Figure 3D). Western blot analysis showed that in septic mice heart tissue, cleaved caspase 3 expression was upregulated whereas Bcl-2 was downregulated. However, pretreatment with neferine upregulated Bcl-2 protein expression and downregulated that of cleaved caspase 3 in septic mice heart tissue (Figures 3E–G). LPS treatment caused significantly increased TUNEL-positive cells, which was apparently reduced by neferine pretreatment (Figure 3H,I). Thus, neferine prevented myocardial morphology injury and cardiomyocyte apoptosis in septic mice.

### Neferine Attenuated Cytotoxicity and Apoptosis in LPS-Treated H9c2 Cells

**FIGURE 4:**
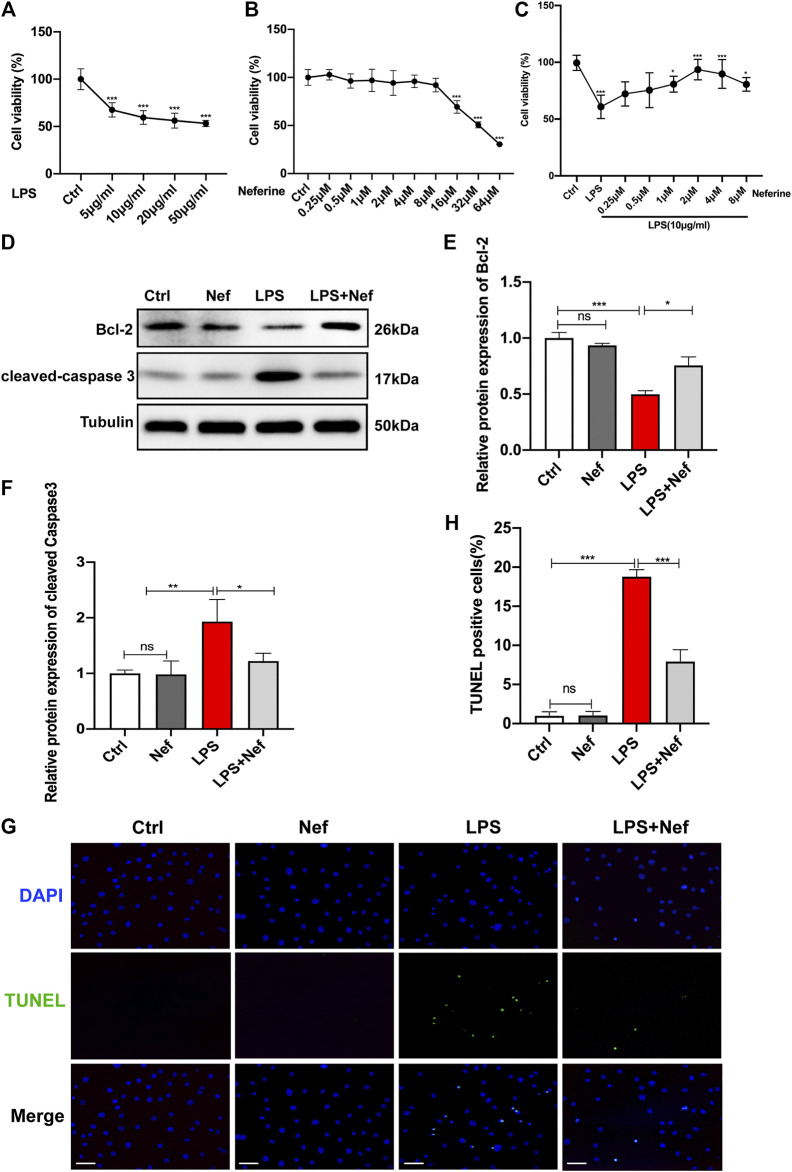
Neferine suppressed apoptosis in LPS-treated H9c2 cells **(A)** Cell Counting Kit-8 Assay (CCK8) was performed to assess cell viability in H9c2 cells that were treated with a concentration gradient of LPS (5, 10, 20, and 50 μg/ml) for 24 h **(B)** The cytotoxicity of different neferine concentrations (0.25, 0.5, 1, 2, 4, 8, 16, 32, and 64 μM) in H9c2 cells was detected by CCK8 assay **(C)** Pretreatment with neferine (0.25, 0.5, 1, 2, 4, and 8 μM) for 2 h prior to LPS exposure (10 μg/ml) for 12 h; cell viability was tested by CCK8 assay **(D–F)** Western blot analysis and densitometric quantification of the protein expression levels of cleaved caspase 3 and Bcl-2 **(G)** TUNEL staining-positive cells (green) labeled apoptotic cells, DAPI (blue) labeled H9c2 cells nuclei. Scale bar, 100 μm **(H)** Measurement of TUNEL-positive cell ratio. All data are expressed as mean ± standard deviation. All experiments were repeated at least three times. *p < 0.05, **p < 0.01, ***p < 0.001; ns: no significant difference; TUNEL, terminal deoxynucleotidyl transferase dUTP nick end labeling; DAPI, 4′,6-diamidino-2-phenylindole.

The results of the induction of sepsis cardiomyopathy by LPS showed that LPS treatment reduced the viability of H9c2 cells to 60% at 10 μg/ml (*p* < 0.01) (Figure 4A). Therefore, we used this concentration in subsequent experiments. To identify the effects of neferine on cytotoxicity, H9c2 cells were incubated with neferine for 24 h. The CCK 8 assay revealed that neferine had no effect on cell viability at low concentrations (0.25–8 μM) but severely affected cell viability at high concentrations (>16 μM). Thus, a high concentration of neferine has an adverse effect on H9c2 cells (Figure 4B). However, pre-incubation with neferine at 1–8 μM can improve cell viability, whereas 2 μM concentration can significantly improve the viability (93%) of H9c2 cells treated with LPS (10 μg/ml) (Figure 4C). Therefore, 2 μM neferine was used in subsequent experiments. Next, we evaluated the effect of neferine on apoptosis in LPS-induced H9c2 cells. Western blot analysis revealed that cleaved caspase 3 expression was upregulated, whereas Bcl-2 expression was downregulated in the LPS group. However, neferine treatment (LPS + Nef) upregulated Bcl-2 protein expression and downregulated cleaved caspase3 expression in LPS-induced H9c2 cells. We preincubated H9c2 cells with neferine for 24 h with no LPS stimulation to eliminate the influence of confounding factors on the results. The Nef group showed no significant differences from the Ctrl group (Figures 4D–F). TUNEL staining showed that neferine significantly reduced the ratio of TUNEL-positive cells in LPS-induced H9c2 cells (Figure 4G,H). These data indicate that neferine exhibited protective effects against LPS-induced apoptosis *in vitro*.

### Neferine Mitigated Mitochondrial Damage and Inhibited the Production of Reactive Oxygen Species In Vivo and In Vitro

**FIGURE 5:**
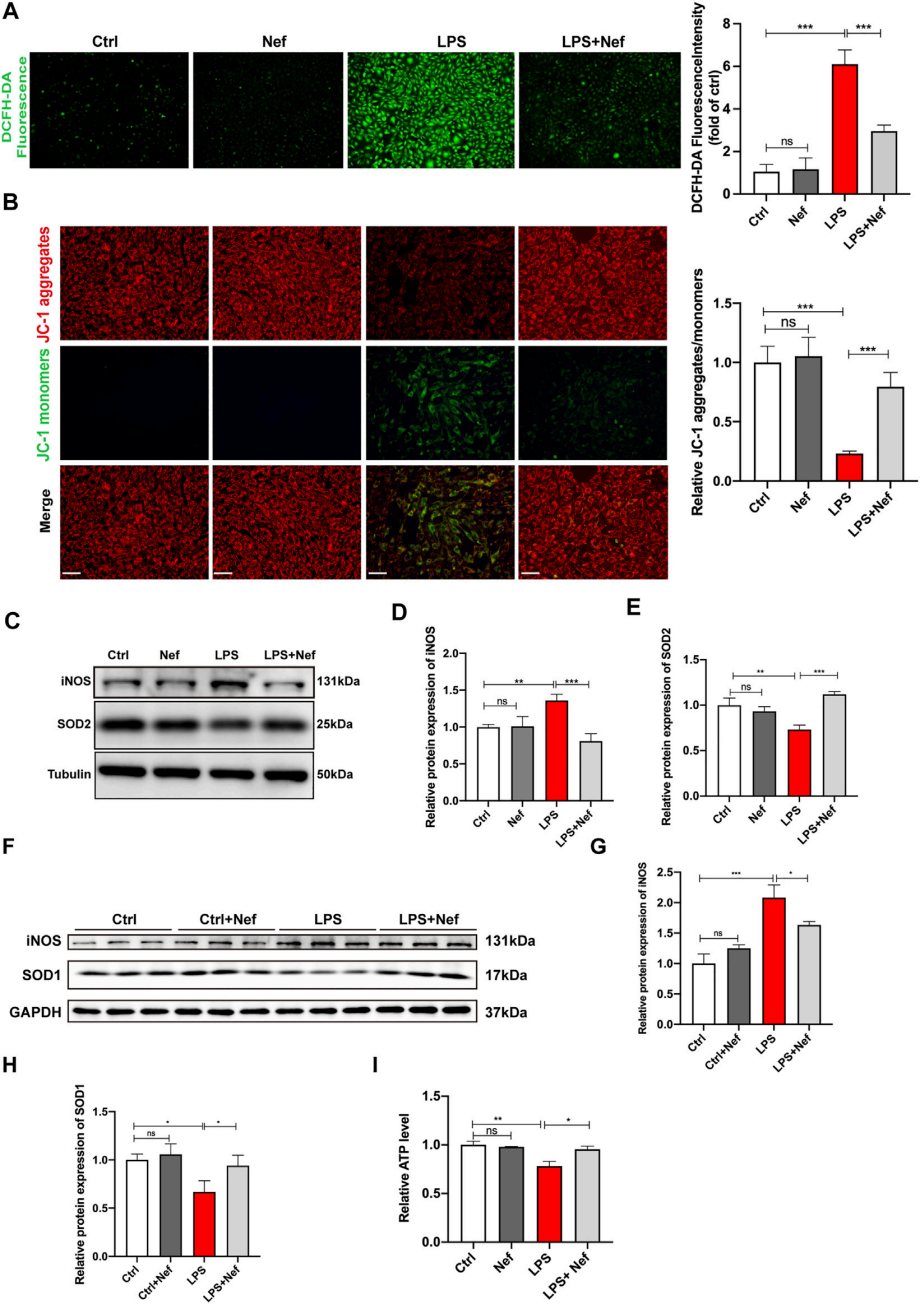
Neferine reduced the production of reactive oxygen species (ROS) and prevented mitochondrial dysfunction **(A)** DCFH-DA staining was used to evaluate the intracellular ROS level in H9c2 cells. Fluorescence intensity was measured. Scale bar, 50 μm **(B)** Representative images of JC-1 staining in LPS-induced H9c2 cells. Fluorescence intensity was measured. Scale bar, 50 μm **(C–E)** Western blot analysis and densitometric quantification of SOD2 and iNOS protein expression in H9c2 cells **(F)** SOD1 and iNOS protein expression levels in septic mice were detected by Western blot (n = 6) **(G, H)** Densitometric quantification of SOD1 and iNOS protein expression levels **(I)** ATP levels in H9c2 cells were analyzed. All data are expressed as mean ± SD. All experiments were repeated at least three times. *p < 0.05, **p < 0.01, ***p < 0.001; ns: no significant difference; DCFH-DA, 2′-7′dichlorofluorescein diacetate.

In the evaluation of intracellular ROS generation in H9c2 cells, DCFH-DA staining revealed that the ROS level was higher in the LPS group than in the Ctrl group. Pre-incubation with neferine (LPS + Nef) reduced ROS generation after LPS treatment, and no significant difference was found between the Ctrl and Nef groups (Figure 5A). The analysis of the mitochondrial function in LPS-induced H9c2 cells demonstrated that the MMP decreased with LPS treatment. This adverse effect was reversed by neferine pretreatment. Conversely, the Nef group did not show any significant difference in MMP from the Ctrl group (Figure 5B). Western blot analysis showed that SOD2 protein expression was downregulated, whereas iNOS protein expression was upregulated in the LPS treatment group. However, pre-culture with neferine (LPS + Nef) upregulated SOD2 protein expression but downregulated iNOS protein expression in LPS-induced H9c2 cells. No significant difference was found between the Ctrl and Nef groups (Figures 5C–E). Consistent with the findings, SOD1 protein expression was downregulated, whereas iNOS protein expression was upregulated in septic mice (LPS group). Neferine pretreatment (LPS + Nef) could upregulate SOD1 protein expression and downregulate iNOS protein expression in the heart tissues of septic mice (Figures 5F–H). Next, ATP assay was applied after Nef + LPS treatment. As shown in Figure 5I, LPS markedly decreased the ATP level, but neferine pretreatment partly reversed this in LPS-induced H9c2 cells. These data suggest that the protective effect of neferine involved the alleviation of mitochondrial dysfunction.

### Neferine Activated the PI3K/AKT/mTOR Signaling Pathway In Vivo and In Vitro

**FIGURE 6:**
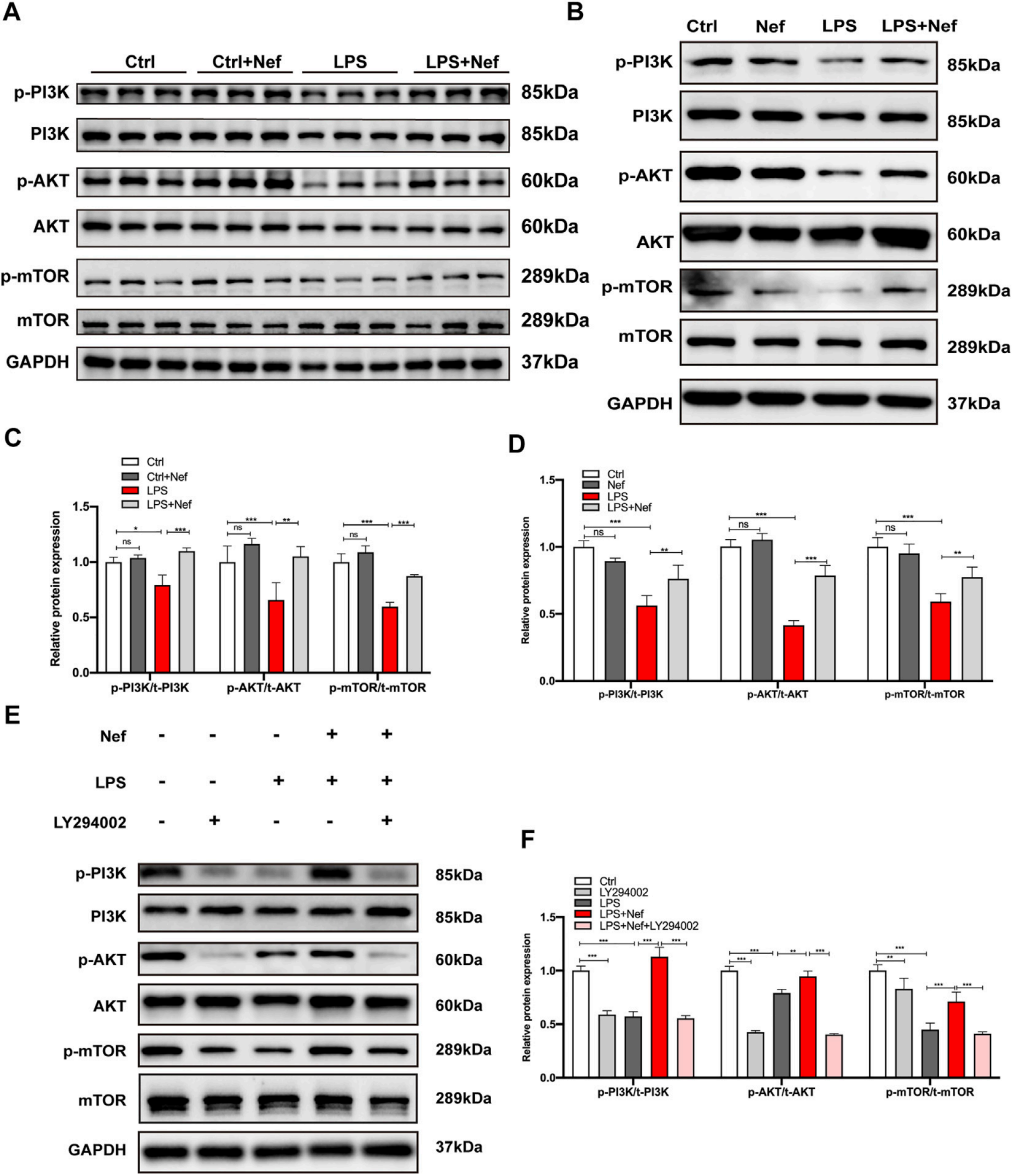
Neferine reversed the LPS-induced downregulation of the PI3K/AKT/mTOR signaling pathway *in vivo* and *in vitro* **(A)** Representative Western blot images of p-PI3K, PI3K, p-AKT, AKT, p-mTOR, and mTOR in mice **(B–E)** Representative Western blot images of p-PI3K, PI3K, p-AKT, AKT, p-mTOR, and mTOR in H9c2 cells **(C)** Densitometric quantification analysis of the protein expression levels of p-PI3K, PI3K, p-AKT, AKT, p-mTOR, and mTOR in mice **(D–F)** Densitometric quantification analysis of the protein expression levels of p-PI3K, PI3K, p-AKT, AKT, p-mTOR, and mTOR in H9c2 cells. All data are expressed as mean ± standard deviation. All experiments were repeated at least three times. *p < 0.05, **p < 0.01, ***p < 0.001; ns: no significant difference.

The PI3K/AKT/mTOR signaling pathway mainly modulates intracellular signal transduction and various biological processes such as inflammation, cell proliferation, apoptosis, metabolism, and angiogenesis and is one of the main signal targets of neferine administration. Western blot analysis revealed that the expression levels of phosphorylated p-PI3K, p-AKT, and p-mTOR protein were downregulated in the LPS group. However, neferine pretreatment (LPS + Nef group) upregulated the expression level of these proteins *in vivo* and *in vitro*. (Figures 6A–D). Thus, neferine administration restored the phosphorylation status of PI3K, AKT, and mTOR *in vivo* and *in vitro*. To further investigate the relative signal mechanisms of neferine in LPS-induced H9c2 cells apoptosis, the PI3K/AKT inhibitor LY294002 was added. The relative expression levels of p-PI3K, p-AKT, and p-mTOR proteins in neferine with LY294002 (LPS + Nef + LY294002 group) were remarkably decreased (Figure 6E,F). Thus, LY294002 could reverse the protective effect of neferine on the PI3K/AKT/mTOR signaling pathway.

### LY294002 Reversed the Protective Effect of Neferine on Apoptosis In Vitro

**FIGURE 7:**
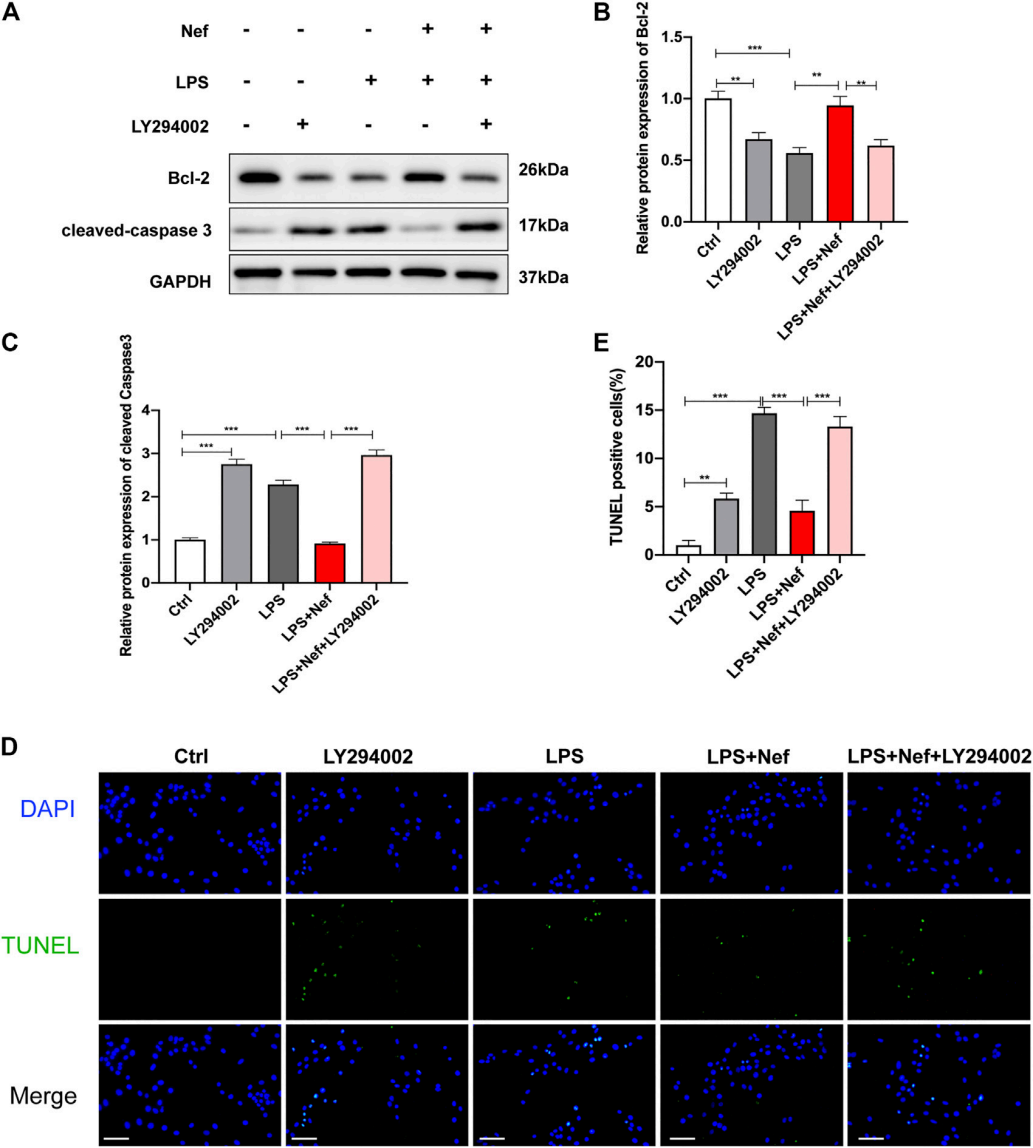
LY294002 impeded the protective effect of neferine on LPS-induced apoptosis in H9c2 cells **(A–C)** Western blot analysis and densitometric quantification of the protein expression levels of cleaved caspase 3 and Bcl-2 **(D)** Representative images of TUNEL staining in H9c2 cells **(E)** Quantification of TUNEL-positive cells. Scale bar, 100 μm. All data are expressed as mean ± standard deviation. All experiments were repeated at least three times. *p < 0.05, **p < 0.01, ***p < 0.001; ns: no significant difference.

Bcl-2 protein is associated with mitochondria-dependent apoptotic signaling. Therefore, the expression levels of Bcl-2 and cleaved caspase 3 were detected by Western blotting. Pre-incubation with neferine restored Bcl-2 expression and reduced cleaved caspase 3 expression in LPS-induced H9c2 cells, but LY294002 reversed this protective effect (Figures 7A–C). TUNEL staining showed that the ratio of TUNEL-positive cells in the LPS + Nef + LY294002 group were remarkably increased compared with that in the LPS + Nef group (Figure 7D,E). These results demonstrate that LY294002 can reverse the protective effects of neferine on H9c2 cell apoptosis.

### LY294002 Reversed the Protective Effect of Neferine on Mitochondrial Dysfunction In Vitro

**FIGURE 8:**
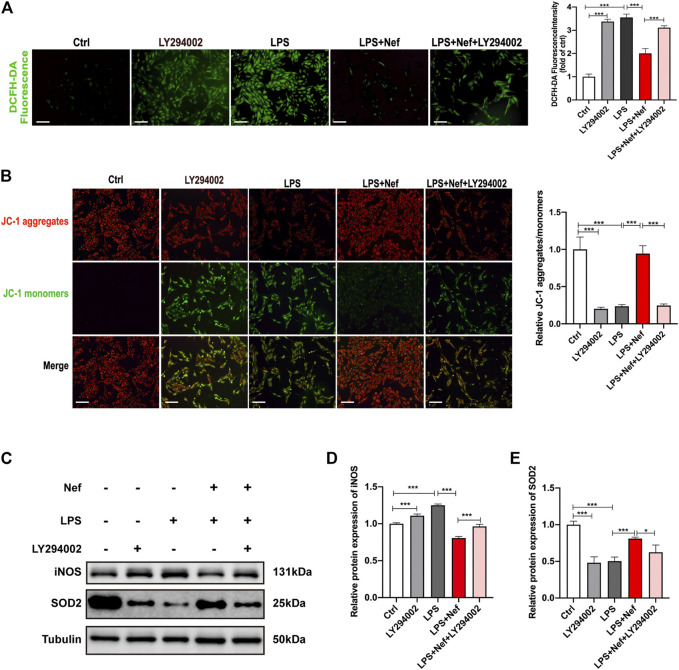
LY294002 inhibited the protective effect of neferine against LPS-induced mitochondrial dysfunction in H9c2 cells **(A)** DCFH-DA staining was used to evaluate the intracellular reactive oxygen species (ROS) level. Fluorescence intensity was measured. Scale bar, 50 μm **(B)** Representative images of JC-1 staining in H9c2 cells. Fluorescence intensity was measured. Scale bar, 50 μm **(C–E)** Western blot analysis and densitometric quantification of the protein expression levels of SOD2 and iNOS. All data are expressed as mean ± standard deviation. All experiments were repeated at least three times. *p < 0.05, **p < 0.01, ***p < 0.001; ns: no significant difference; DCFH-DA, 2′-7′dichlorofluorescein diacetate.

DCFH-DA staining revealed that the ROS level was higher in the LPS + Nef + LY294002 group than in the LPS + Nef group (Figure 8A). Likewise, the mitochondrial JC-1 assay demonstrated that the ROS level decreased with LPS treatment. This adverse effect was restored by neferine treatment (LPS + Nef group) but worsened by LY294002 treatment (LPS + Nef + LY294002) (Figure 8B). Western blot analysis showed that SOD2 protein expression was downregulated, whereas iNOS protein expression was upregulated in the LPS + Nef + LY294002 group (Figures 8C–E). Thus, LY294002 reverses the protective role of neferine on mitochondrial dysfunction in LPS-induced H9c2 cells.

## Discussion

Overall, our study demonstrates that neferine has a protective effect against LPS-induced myocardial dysfunction. *In vivo*, neferine improved survival rate and cardiac function and reduced myocardial pathological damage and myocardial cell apoptosis. *In vitro*, neferine alleviated the apoptosis of H9c2 cells treated with LPS, inhibited ROS production, and improved mitochondrial function. In addition, the data show that neferine activated the PI3K/AKT/mTOR signaling pathway. Conversely, the PI3K/AKT pathway inhibitor LY294002 reversed the inhibitory effect of neferine on LPS-induced apoptosis in H9c2 cells. Therefore, this study provides evidence that neferine ameliorates LPS-induced myocardial dysfunction through anti-apoptosis and antioxidative stress by regulating the PI3K/AKT/mTOR pathway.

The protection of heart function is pivotal in the occurrence and development of sepsis. Cardiomyocyte damage and cell death caused by sepsis are mainly mediated by the body’s detection and response to LPS (Pfalzgraff and Weindl, 2019). Blocking LPS-induced apoptosis may increase cell viability, reduce oxidative stress, and improve mitochondrial damage (Han et al., 2020; Xu et al., 2020). Therefore, finding key compounds resistant to LPS-induced cell apoptosis is very important for protecting cardiomyocytes. The heart is a major source of hemodynamic forces in the human body, and mitochondria are the primary source of energy supply in the heart. Mitochondria play a vital role in maintaining the homeostasis of cardiomyocytes, which could be disrupted by excessive ROS. Accumulating evidence demonstrates that septic cardiomyopathy involves mitochondrial dysfunction (Chen et al., 2019; Stanzani et al., 2019; Joseph et al., 2020). Mitochondrial ROS are the driving force of mitochondrial dysfunction in septic cardiomyopathy (Joseph et al., 2020). Studies have found that in septic cardiomyopathy, mitochondrial dysfunction is a key contributor to impaired mitochondrial function and increased mitochondrial fission, leading ultimately to mitochondrial dysfunction and cardiomyocyte apoptosis (Haileselassie et al., 2019). When mitochondria are attacked, excessive ROS are released, and lipid molecules, proteins, and DNA are rapidly destroyed by oxygen free radicals, which eventually lead to cell death (Joseph et al., 2017).

Studies have demonstrated that free radicals cause loss of membrane integrity and lipid membrane peroxidation (Jing et al., 2015). Therefore, various myocardial enzymes are released from damaged tissues into the circulation (Hu et al., 2015). The physiological activities exerted by SODs include antioxidative and anti-inflammatory effects (Güleç et al., 2006). SOD2 is the primary antioxidant enzyme found in mitochondria, which can neutralize oxygen free radicals (Zhang et al., 2017). On the contrary, iNOS is a critical enzyme involved in the progression of inflammation and oxidative dysfunction (You et al., 2013). Evidence has indicated that SOD2 and iNOS are closely involved in LPS-induced myocardial dysfunction (Tsai et al., 2012; Yang et al., 2013). Our findings suggest that neferine pretreatment reduced ROS levels and iNOS protein expression and improved mitochondrial function by upregulating MMP and the expression lof SOD2 and SOD1. In addition, neferine pretreatment also improved ATP levels.

Next, we explored the potential mechanisms by which neferine protects against LPS-induced myocardial injury. PI3K/AKT/mTOR is a classic signaling pathway that regulates intracellular processes, including cell survival, metabolism, proliferation, differentiation, and apoptosis (Franke et al., 1997; Vanhaesebroeck et al., 2012). Recent studies have shown that autophagy and apoptosis play a vital role in the cell survival signaling pathway and that PI3K/AKT/mTOR signaling regulates autophagy (Poornima et al., 2013). Chen et al. (2017) demonstrated that PI3K/AKT/mTOR signaling was significantly changed in sepsis myocardial injury, whereas Shang et al. (2019) found that resveratrol protected the myocardium in septic rats by activating the PI3K/AKT/mTOR signaling pathway and inhibiting the NF-κB signaling pathway and related inflammatory factors. However, the mechanism related to cell apoptosis is still uncertain in LPS-induced cardiac dysfunction. Our study found that neferine increased p-PI3K, p-AKT, and p-mTOR levels. In addition, neferine reversed LPS-induced decreases in PI3K, AKT, and mTOR phosphorylation, which indicates that neferine activates the PI3K/AKT/mTOR signaling pathway *in vivo* and *in vitro*. These findings are in accordance with the previous observation that neferine upregulates the PI3K/AKT/mTOR signaling pathway in doxorubicin-treated H9c2 cardiomyoblasts (Bharathi Priya et al., 2018).

To confirm that neferine indeed regulated LPS-induced H9c2 cells apoptosis through the PI3K/AKT/mTOR signaling pathway, we introduced the PI3K-specific inhibitor LY294002. The levels of apoptosis-related proteins and TUNEL-positive cells were assessed. The results showed that LY294002 reversed the protective effect of neferine on LPS-induced H9c2 cell apoptosis. In addition, we found that neferine treatment reduced ROS production, increased mitochondrial membrane potential, and preserved mitochondrial function. However, LY294002 reversed the protective effects of neferine on mitochondrial function *in vitro*. The results showed that the PI3K/AKT/mTOR pathway was involved in the protective role of neferine against LPS-induced myocardial injury by regulating the expression of apoptosis- and oxidation-related proteins. These proteins play an important role in inhibiting cell apoptosis, reducing oxidative stress, and thus alleviating septic cardiomyopathy. However, further research is still needed to explore the specific mechanisms involving neferine in regulating the PI3K/AKT/mTOR pathway.

**FIGURE 9:**
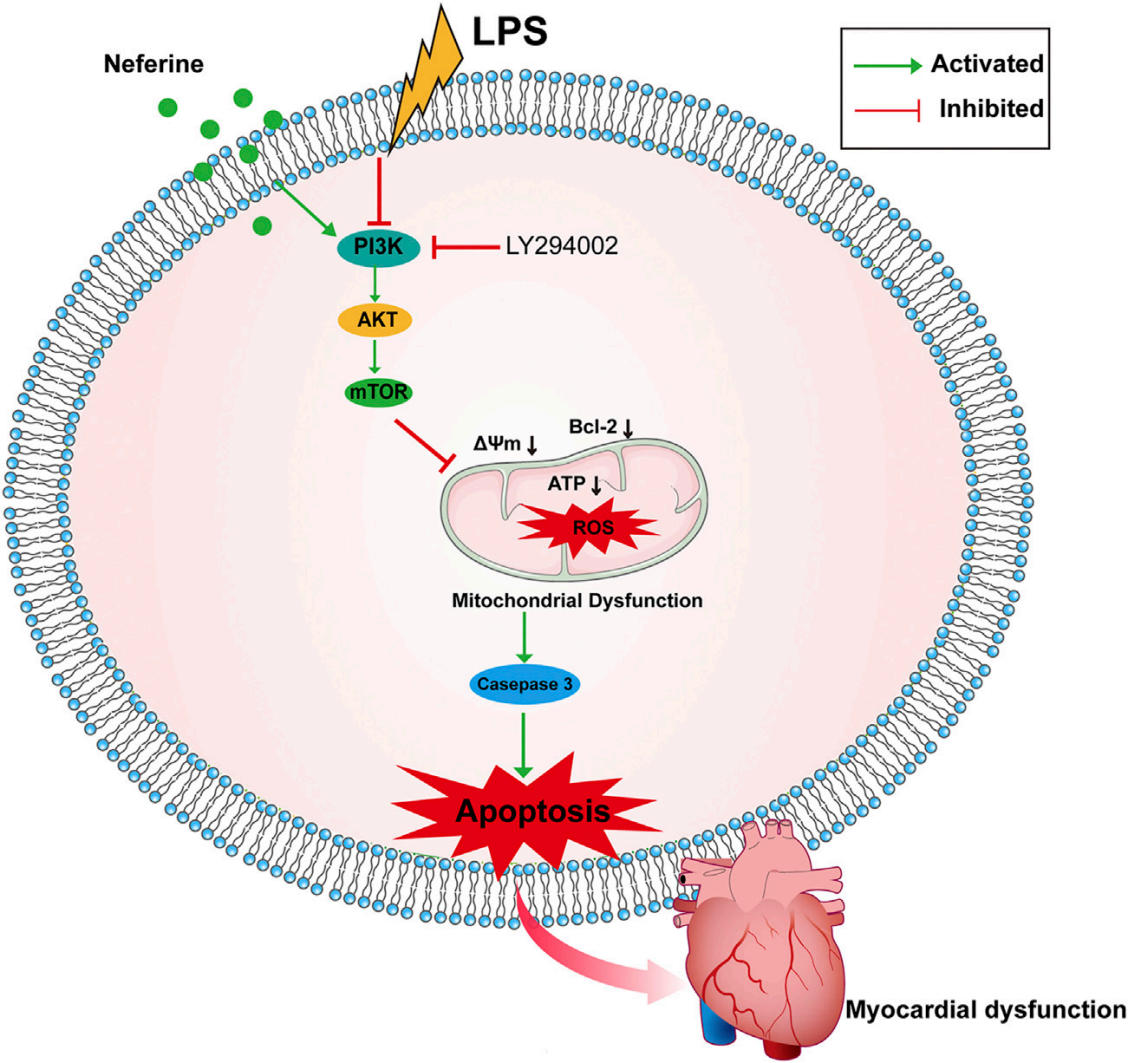
Schematic representation of the effects of neferine on sepsis-induced myocardial dysfunction. Neferine suppressed LPS-induced cells apoptosis by regulating the PI3K/AKT/mTOR signaling pathway, which attenuates oxidative damage.

This study provides preliminary evidence that neferine exerts protective effects against LPS-induced cardiac dysfunction *in vivo* and *in vitro*. Neferine suppressed cell apoptosis and oxidative damage via the PI3K/AKT/mTOR signaling pathway (Figure 9). Consequently, neferine may be a promising therapeutic strategy for preventing sepsis-related myocardiopathy.

## Data Availability

The raw data supporting the conclusion of this article will be made available by the authors, without undue reservation.
