# Corrigendum: Neferine Ameliorates Sepsis-Induced Myocardial Dysfunction Through Anti-Apoptotic and Antioxidative Effects by Regulating the PI3K/AKT/mTOR Signaling Pathway

**DOI:** 10.3389/fphar.2022.913778

**Published:** 2022-04-29

**Authors:** Zhen Qi, Renrong Wang, Rongheng Liao, Song Xue, Yongyi Wang

**Affiliations:** ^1^ Department of Cardiovascular Surgery, Renji Hospital, School of Medicine, Shanghai Jiao Tong University, Shanghai, China; ^2^ Department of Cardiology, Wuxi No. 2 Hospital, Nanjing Medical University, Wuxi, China

**Keywords:** neferine, apoptosis, oxidative stress, mitochondria, lipopolysaccharide, cardiac dysfunction, sepsis

In the original article, there was a mistake in the caption for Figure 6C as published. A clerical error occurred when we prepared the paper. The correct caption appears below.

“(C) Densitometric quantification analysis of the protein expression levels of p-PI3K, PI3K, p-AKT, AKT, p-mTOR, and mTOR in mice.”

In the original article, there was a mistake in Figures 2, 5, 6 as published. The image in the “LPS + Nef” group within Figure 2B, the image in the “LPS + Nef” group within Figure 5A, and the mTOR bands within Figure 6E, were uploaded with errors. The corrected Figures 2, 5, 6 appear below.

**FIGURE 2:**
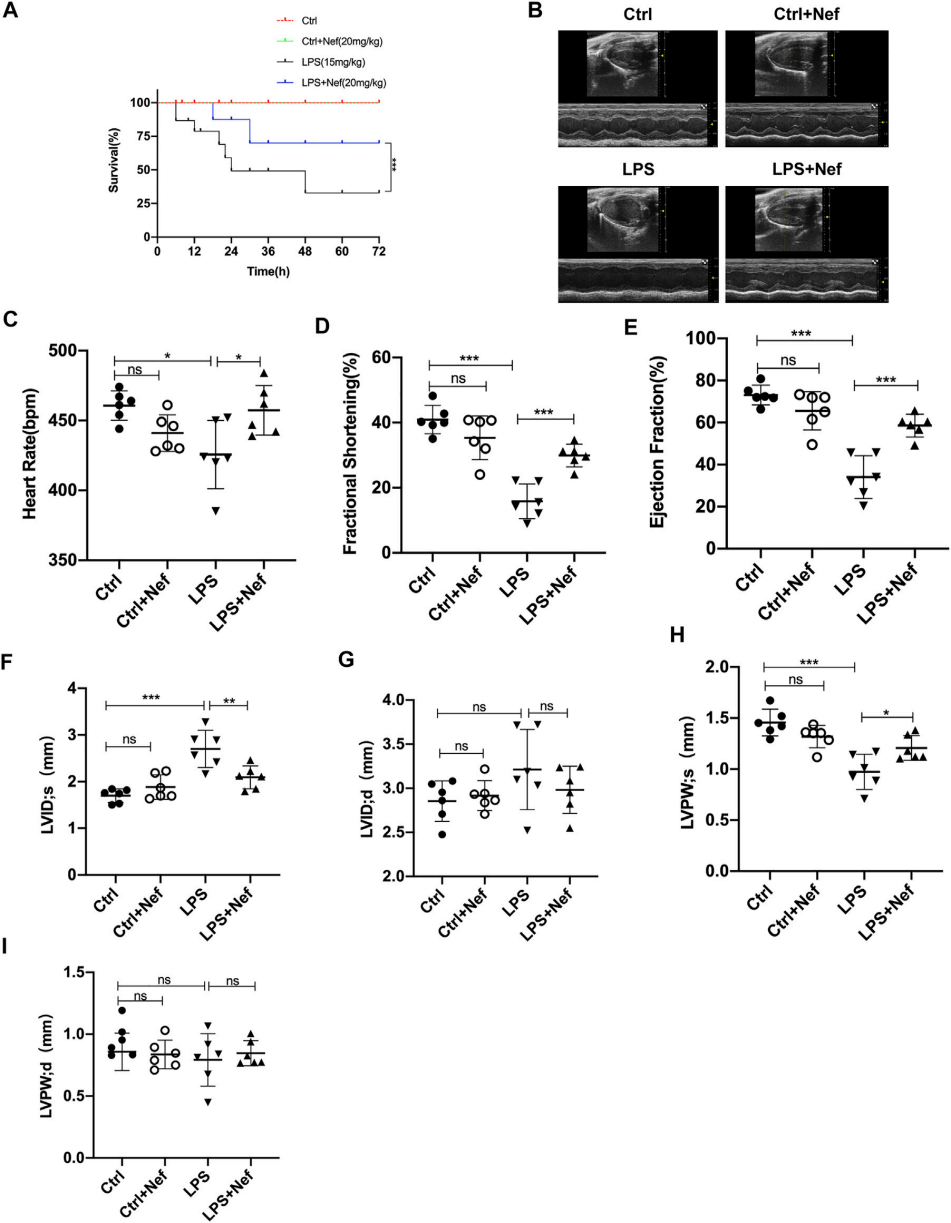
Neferine preserved cardiac function and improved the survival rate in LPS-treated mice. **(A)** Neferine (20 mg/kg) was intraperitoneally administered 2 h before LPS injection (15 mg/kg) and then administered for three consecutive days. The mortality of mice within 72 h was recorded (*n* = 15 mice). **(B–I)** the mice were treated with neferine (20 mg/kg, intraperitoneally (i.p.) 2 h before LPS challenge (10 mg/kg, i.p.), and cardiac function was examined (*n* = 6). **(B)** Representative echocardiographic images. **(C)** Heart rate (HR). **(D)** Fractional shortening (FS). **(E)** Ejection fraction (EF). **(F)** Left ventricular internal systolic dimension (LVIDs). **(G)** Left ventricular internal diastolic dimension (LVIDd). **(H)** Left ventricular posterior wall systolic thickness (LVPWs). **(I)** Left ventricular posterior wall diastolic thickness (LVPWd). Data are expressed as mean ± standard deviation. *p < 0.05, **p < 0.01, ***p < 0.001; ns: no significant difference.

**FIGURE 5:**
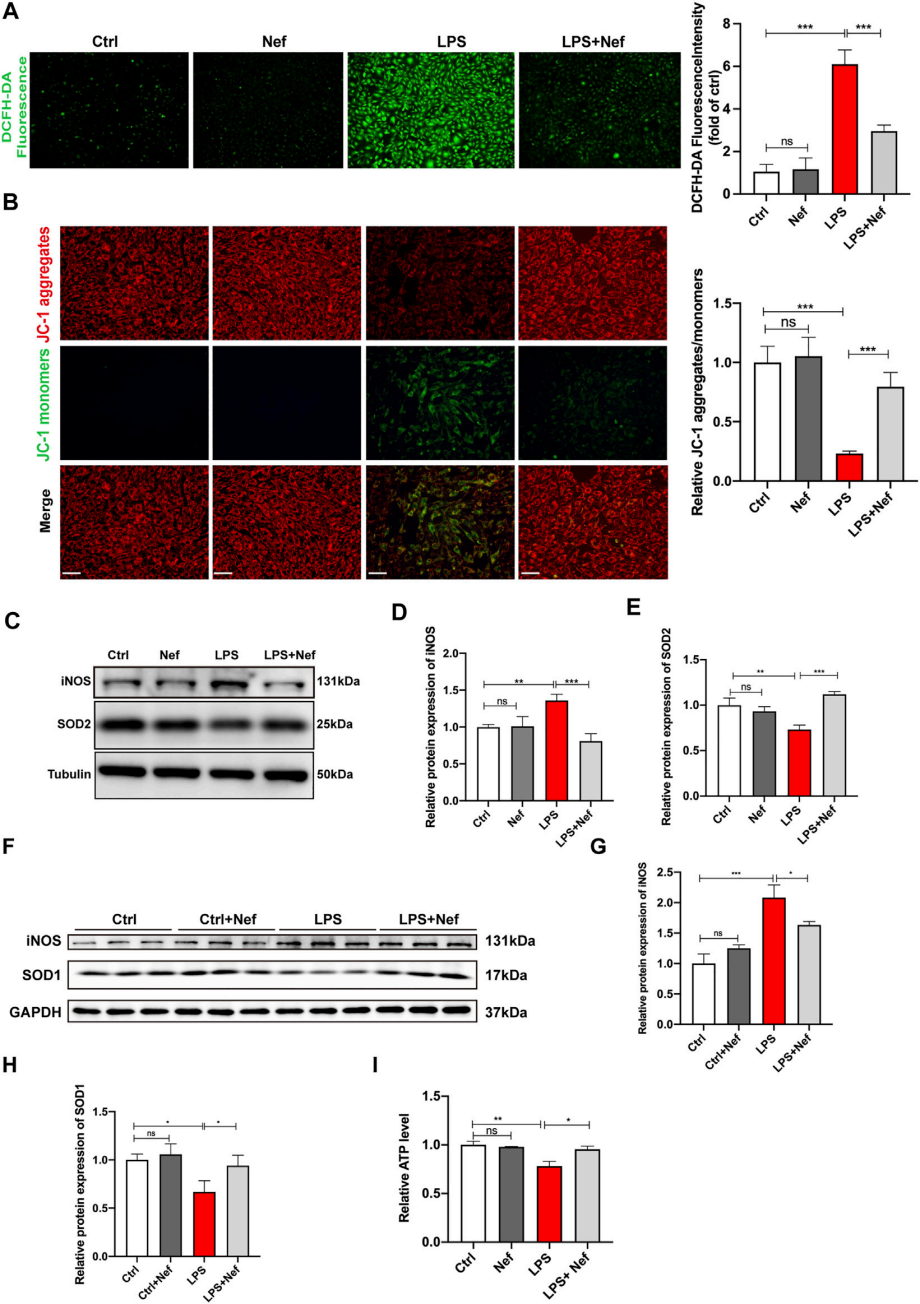
Neferine reduced the production of reactive oxygen species (ROS) and prevented mitochondrial dysfunction. **(A)** DCFH-DA staining was used to evaluate the intracellular ROS level in H9c2 cells. Fluorescence intensity was measured. Scale bar, 50 μm. **(B)** Representative images of JC-1 staining in LPS-induced H9c2 cells. Fluorescence intensity was measured. Scale bar, 50 μm. **(C–E)** Western blot analysis and densitometric quantification of SOD2 and iNOS protein expression in H9c2 cells. **(F)** SOD1 and iNOS protein expression levels in septic mice were detected by Western blot (*n* = 6). **(G,H)** Densitometric quantification of SOD1 and iNOS protein expression levels. **(I)** ATP levels in H9c2 cells were analyzed. All data are expressed as mean ± SD. All experiments were repeated at least three times. *p < 0.05, **p < 0.01, ***p < 0.001; ns: no significant difference; DCFH-DA, 2′-7′dichlorofluorescein diacetate.

**FIGURE 6:**
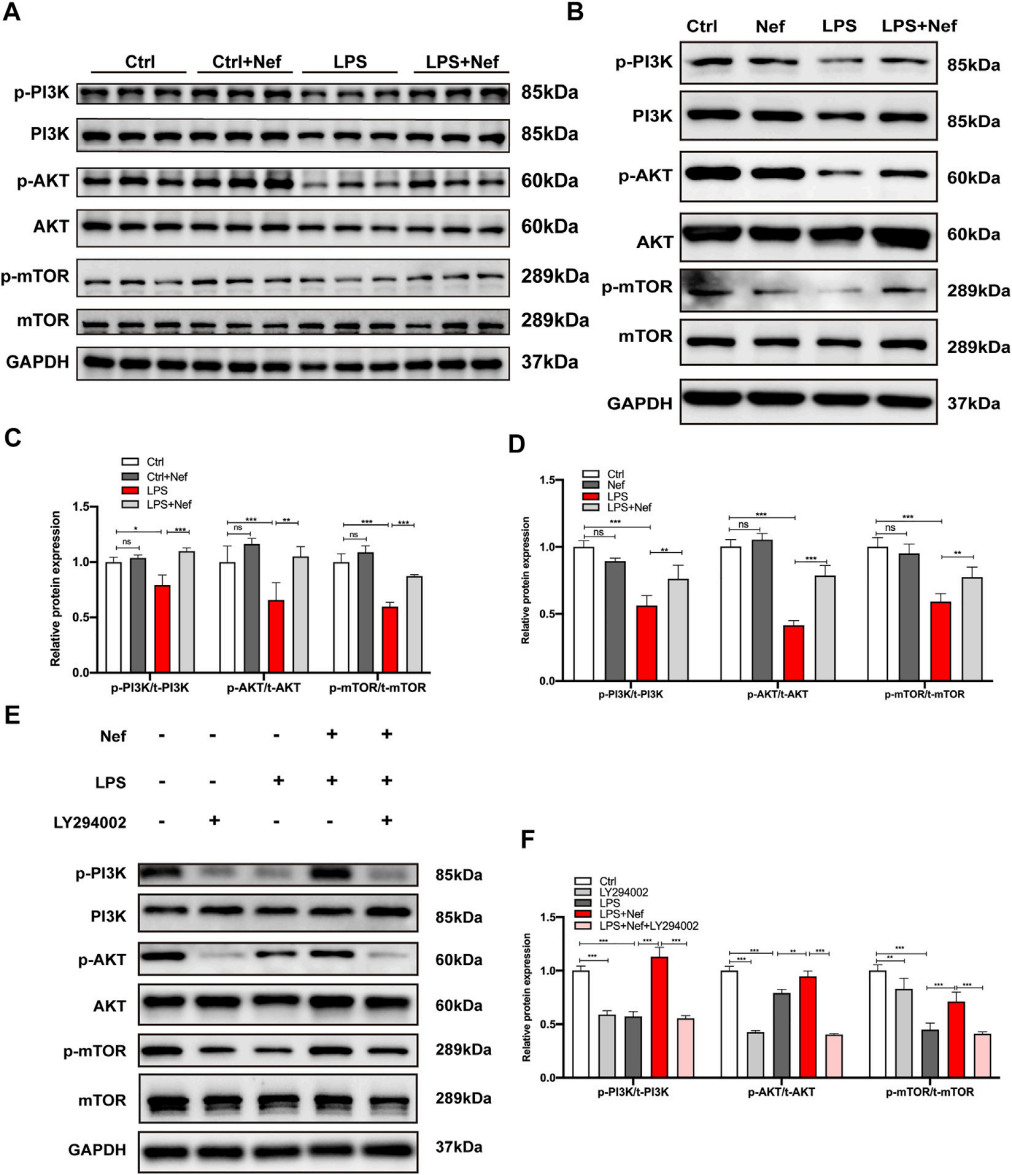
Neferine reversed the LPS-induced downregulation of the PI3K/AKT/mTOR signaling pathway *in vivo* and *in vitro*. **(A)** Representative Western blot images of p-PI3K, PI3K, p-AKT, AKT, p-mTOR, and mTOR in mice. **(B–E)** Representative Western blot images of p-PI3K, PI3K, p-AKT, AKT, p-mTOR, and mTOR in H9c2 cells. **(C)** Densitometric quantification analysis of the protein expression levels of p-PI3K, PI3K, p-AKT, AKT, p-mTOR, and mTOR in mice. **(D–F)** Densitometric quantification analysis of the protein expression levels of p-PI3K, PI3K, p-AKT, AKT, p-mTOR, and mTOR in H9c2 cells. All data are expressed as mean ± standard deviation. All experiments were repeated at least three times. *p < 0.05, **p < 0.01, ***p < 0.001; ns: no significant difference.

The authors apologize for this error and state that this does not change the scientific conclusions of the article in any way. The original article has been updated.

